# Experimental and 3D Simulation Research on the Mechanical Properties of Cold-Bonded Fly Ash Lightweight Aggregate Concrete Exposed to Different High Temperatures

**DOI:** 10.3390/ma18214991

**Published:** 2025-10-31

**Authors:** Shuai Xu, Pengfei Fu, Yanyan Liu, Ting Huang, Xiuli Wang, Yan Li

**Affiliations:** 1 School of Civil Engineering, Jilin Jianzhu University, Changchun 130118, China; fupengfei@student.jlju.edu.cn (P.F.); wangxiuli@jlju.edu.cn (X.W.); liyan@jlju.edu.cn (Y.L.); 2 Key Laboratory of Architectural Cold Climate Energy Management, Ministry of Education, Jilin Jianzhu University, Changchun 130118, China; 3 National Engineering Research Center of Biomaterials, Nanjing Forestry University, Nanjing 210037, China; liuyanyan@njfu.edu.cn; 4 Guangxi Key Laboratory of Green Building Materials and Construction Industrialization, Guilin University of Technology, Guilin 541004, China; tingingh@glut.edu.cn

**Keywords:** cold-bonded, fly ash lightweight aggregate concrete, performance after high temperature, three-dimensional mesoscale model, compressive strength, fire resistance

## Abstract

Cold-bonded (CB) fly ash aggregate, an eco-friendly material derived from industrial by-products, is used to fully replace natural coarse aggregate in producing lightweight concrete (LWC-CB). This study systematically investigates the post-high-temperature mechanical properties and damage mechanisms of LWC-CB. Specimens exposed to ambient temperature (10 °C) and elevated temperatures (200 °C, 400 °C, 600 °C) underwent cubic compression tests, with surface deformation monitored via digital image correlation (DIC). Experimental results indicate that the strength retention of LWC-CB is approximately 6% superior to ordinary concrete below 500 °C, beyond which its performance converges. Damage analysis reveals a transition in failure mode: at ambient temperature, shear failure is governed by the low intrinsic strength of CB aggregates, while after high-temperature exposure, damage localizes within the mortar and the interfacial transition zone (ITZ) due to mortar micro-cracking and thermal mismatch. To elucidate these mechanisms, a three-dimensional mesoscale model was developed and validated, effectively characterizing the internal multiphase structure at room temperature. Furthermore, a homogenization model was established to analyze the macroscopic thermo-mechanical response. The numerical simulations show strong agreement with experimental data, with a maximum deviation of 15% at 10 °C and 3% after high-temperature exposure, confirming the model’s accuracy in capturing the performance evolution of LWC-CB.

## 1. Introduction

The deadweight of a concrete structure accounts for a significant portion of the total structural load. Therefore, reducing the density of concrete offers obvious advantages for structural efficiency [1]. In China, the definition of lightweight aggregate concrete is as follows: a type of concrete prepared with lightweight coarse aggregates, lightweight sand or ordinary sand, cementitious materials, admixtures, and water, with a dry bulk density not exceeding 1950 kg/m^3^. Lightweight aggregate concrete, with its significant practical value and economic benefits, has become one of the important materials in the field of modern construction, while also demonstrating good applicability and application potential in roadway structural layers [2]. Concurrently, the management of industrial waste remains a critical environmental issue. For instance, more than 100 million tonnes of fly ash are stockpiled every year because it cannot be used in a timely manner in China [3]. Concrete technology opens a new path for the recycling of such industrial waste [4,5,6,7,8]. Specifically, the use of fly ash aggregate to replace natural aggregate in concrete production can effectively achieve the reuse of fly ash, which aligns with environmental objectives and promotes sustainable development.

The production of fly ash aggregate is primarily divided into the sintering method and the cold-bonding method. Compared with the traditional sintering process, the cold-bonding method represents a simpler, more energy-saving, and environmentally friendly process for the preparation of ceramic granules. However, when compared to natural aggregates, cold-bonded (CB) fly ash aggregates are characterized by higher water absorption, lower density, and lower intrinsic strength [9].

The damage caused by fire to buildings is systemic, severely testing structures from a mechanical to a functional level [10,11]. High temperatures during a fire induce complex physico-chemical changes within the concrete material [12], which in turn negatively affect its mechanical performance [13]. For instance, the cubic compressive strength of concrete after exposure to high temperatures typically shows an overall decreasing trend as the temperature increases [14,15]. The mechanical properties of concrete are indeed adversely affected when exposed to high temperatures. Initial deterioration begins to occur in the temperature range of 200 °C to 300 °C. This deterioration continues and intensifies as the temperature rises further, leading to a significant reduction in the structural strength and stiffness [16,17].

The mechanical properties of lightweight aggregate concrete after high-temperature exposure differ from those of ordinary concrete due to differences in the properties of its internal coarse and fine aggregates and the cement paste. For example, the post-high-temperature losses in cubic compressive strength, axial compressive strength, and splitting tensile strength are often less for lightweight aggregate concrete than for ordinary concrete. Furthermore, as the water absorption of the lightweight aggregate increases, the loss of compressive strength in specimens after high temperatures is often reduced [18]. Supporting this.

**Figure 1:**
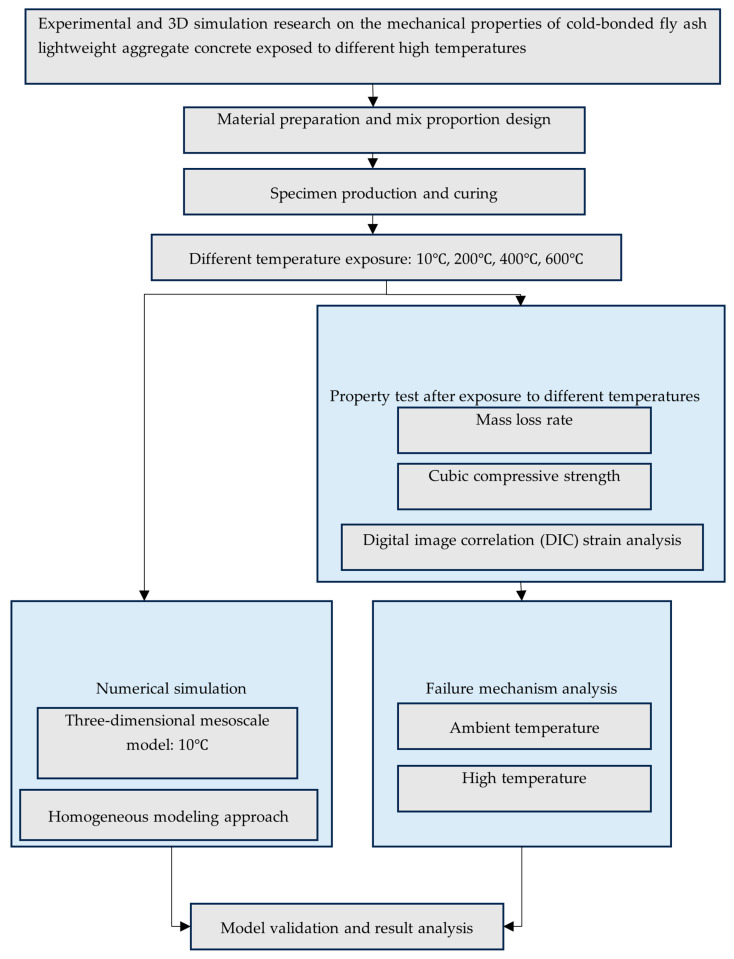
Experimental flowchart.

Guangchao Hu [19] developed a non-fired ceramsite construction material with enhanced lightweight and high-strength properties. Phuong Trinh Bui [20] evaluated the comprehensive performance of cold-bonded aggregates with different amounts of Class F fly ash and Portland cement. The study concluded that cold-bonded aggregates made with 85% fly ash and 15% cement achieved the optimal balance between performance, cost, and environmental sustainability, representing the most favorable choice for sustainable concrete production. The existing literature on the post-fire properties of concrete primarily focuses on natural aggregate concrete and lightweight aggregate concrete utilizing sintered aggregates. In contrast, there are considerably fewer studies investigating lightweight aggregate concrete that uses cold-bonded (CB) fly ash aggregate as a full substitute for natural aggregate. Therefore, this paper addresses this research gap by configuring LWC-CB cubic specimens of strength grade LC25, where CB fully replaces natural aggregates. The mechanical properties and damage mechanism of LWC-CB after exposure to both normal and high temperatures are investigated through experimental tests on cubic specimens at room temperature and elevated temperatures. Additionally, the compression process of the specimen is simulated and analyzed with the aid of finite element software ABAQUS 2022. This work provides an important theoretical basis for the maintenance and safety assessment of related engineering structures after a fire event. The specific experimental flowchart is shown in Figure 1. The list of synonyms involved in the article is explained in Table 1.

## 2. Test Program

### 2.1. Materials

#### 2.1.1. Coarse Aggregate

**Figure 2:**
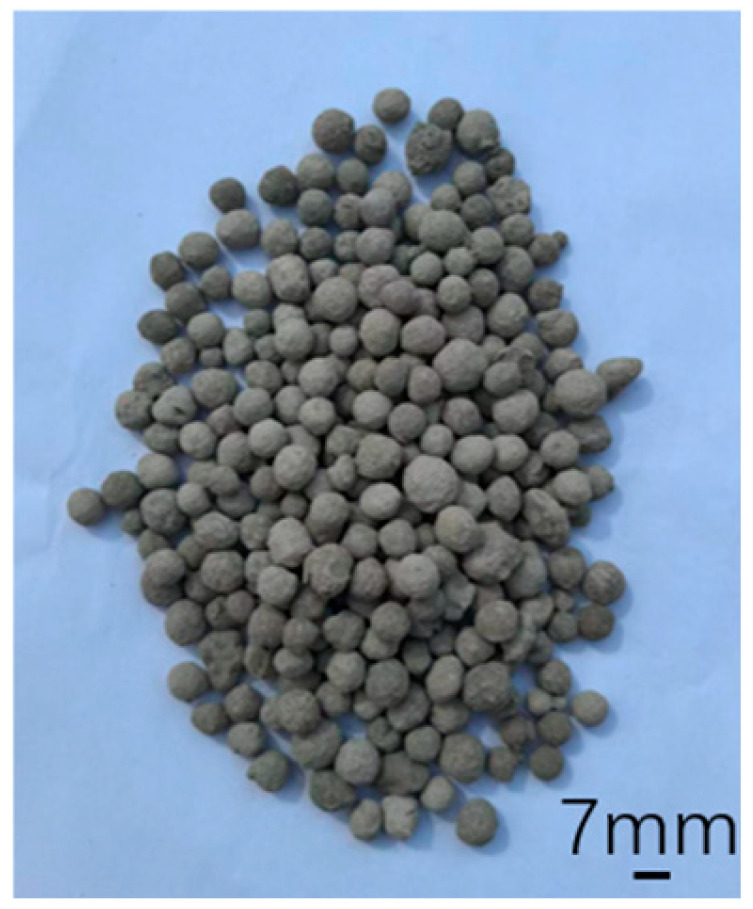
Cold-bonded fly ash aggregate.

The cold-bonded (CB) fly ash aggregate produced by Gongyi Longze Water Purification Materials Co., Ltd. (Gongyi, China) was used for fabricating the test blocks is shown in Figure 2, and its corresponding physical properties are listed in Table 2. The parameters in Table 1 are defined as follows. Packing density: This refers to the mass per unit volume of granular materials in their natural, loosely packed state, which includes the voids between particles. Apparent density: This denotes the mass per unit volume of the material itself, including any internal pores or voids within individual particles, but excluding the interstitial voids between particles. Cylinder compression strength: This is the compressive strength of the ceramsite aggregate as measured by the cylinder pressure method (e.g., according to GB/T 17431.1) [21]. It is specifically defined as the pressure value recorded when a specified proportion of the aggregate particles is crushed within a standardized cylindrical mold.

#### 2.1.2. Fine Aggregate

Ordinary river sand produced by Jilin Yatai Building Materials Group (Changchun, China) was employed as the fine aggregate in this experimental program. Its primary physical properties are detailed in Table 3.

#### 2.1.3. Other Materials

Ordinary Portland cement produced by Jilin Yatai Cement Co., Ltd. (Changchun, China) was used as the primary binder, with fly ash (Gongyi Longze Water Purification Materials Co., Ltd., Gongyi, China) incorporated as a supplementary cementitious material. The chemical compositions of the major components for both the cement and the fly ash are provided in Table 4 and Table 5, respectively.

### 2.2. Cubic Specimen Production

Given the significant variability between different types of ceramic granules and even between batches from different manufacturers, this study based its mix design on the general requirements and relevant recommendations stipulated in the JGJ/T12-2019 ‘Lightweight Aggregate Concrete Application Technical Standards’ [22] for a target strength grade of LC25.

The mix design procedure was as follows: First, the suitable design strength of the concrete was determined using Equation (1):

(1) $$f_{cu,0}\geq f_{cu,k}+1.645\sigma =30+1.645\times 5=33.225\mathrm{MPa}$$

where *f*_cu,0_ is the suitable design strength of the lightweight aggregate concrete (MPa), *f*_cu,k_ is the standard value of the cubic compressive strength (MPa), *σ* is the standard deviation of the lightweight aggregate concrete strength (MPa), taken as 5 based on Table 5.2.2 of the JGJ/T12-2019 standard.

Subsequently, key parameters, including the cement dosage, water–cement ratio, net water consumption, total volume of coarse and fine aggregates (*V*_t_), and the sand rate (*S*_p_), were determined in accordance with the JGJ/T12-2019 standard and the specific properties of the ceramic granules. Following the selection of these parameters, the proportional mix design was carried out using the loose volume method. The mass of fine and coarse aggregates was derived from Equations (2)–(5):

(2) $$V_{s}=V_{t}\times S_{p}$$

(3) $$m_{s}=V_{s}\times \rho_{s}$$

(4) $$V_{a}=V_{t}{-V}_{s}$$

(5) $$m_{a}=V_{a}\times \rho_{a}$$

where *V*_s_ is the volume of fine aggregate in the concrete (m^3^), *m*_s_ is the mass of fine aggregate (kg), *ρ*_s_ is the bulk density of the fine aggregate (kg/m^3^), *V*_a_ is the volume of coarse aggregate in the concrete (m^3^), *m*_a_ is the mass of coarse aggregate (kg), *ρ*_a_ is the bulk density of the coarse aggregate (kg/m^3^).

In summary, the required mass of sand, CB aggregate, cement, and net water per cubic meter was calculated. The total mixing water was then determined by calculating the additional water required to account for the water absorption of the CB aggregate. This process yielded a preliminary mix proportion for LC25 concrete. The water–cement ratio and sand rate were subsequently varied based on the results of initial tests and calculations to arrive at the final, optimized mix ratio for LC25 concrete, which is presented in Table 6.

The 28-day cubic compressive strength of the specimens prepared according to this mix proportion is summarized in Table 7. The real volumetric density of this concrete is 1843 kg/m^3^. According to Chinese standards, this concrete is classified as lightweight aggregate concrete.

### 2.3. High-Temperature Testing Program

**Figure 3:**
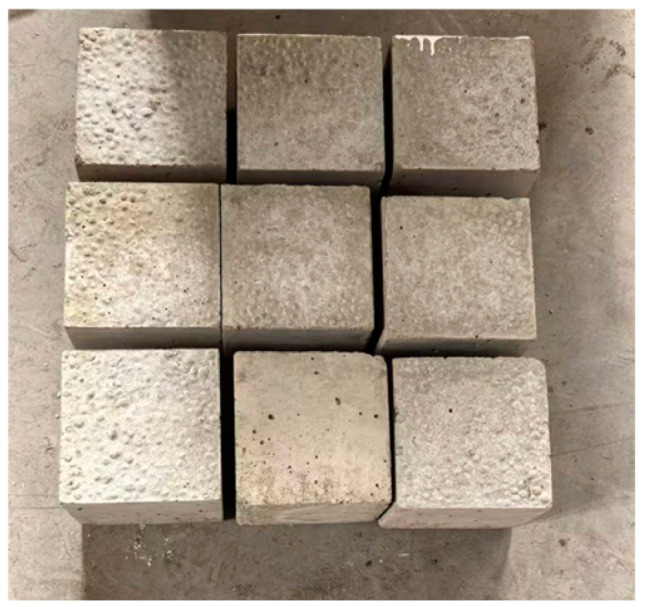
Cubic test block.

Using the finalized mix proportion, nine cubic specimens (150 mm × 150 mm × 150 mm) were prepared, as illustrated in Figure 2. These specimens were subsequently cured under standard conditions for 28 days. Following the curing period, they were removed from the curing environment and air-dried at 10 °C for one month. After this air-drying phase, each specimen was weighed, and its mass was recorded as m_1_. An image of an air-dried test block is presented in Figure 3.

**Figure 4:**
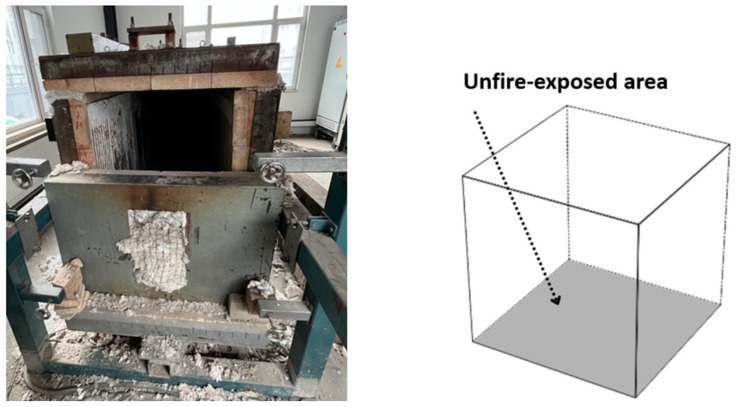
Concrete specimens subjected to high-temperature effect.

**Figure 5:**
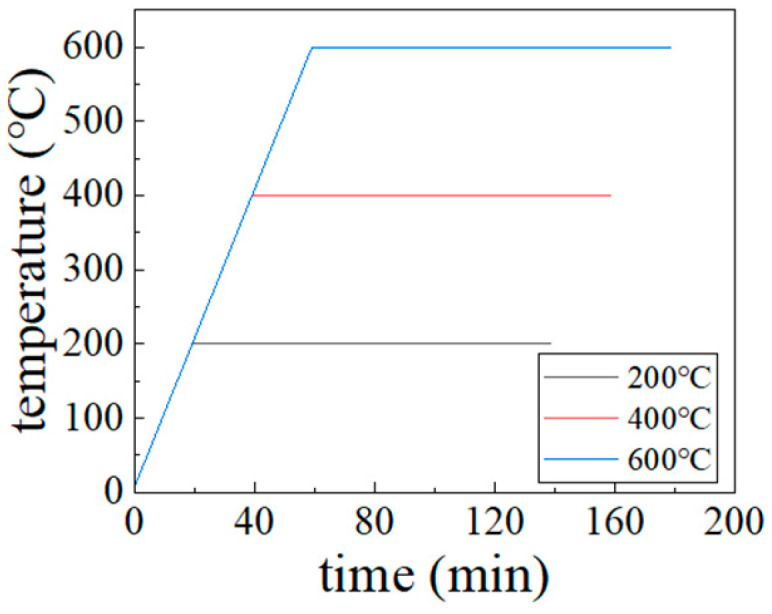
Heating curve.

The experimental heating process was conducted using a laboratory box-type resistance furnace. The test blocks were heated in separate batches to the target high temperatures of 200 °C, 400 °C, and 600 °C. The heating setup is illustrated in Figure 4. In this configuration, only the bottom surface of each block was shielded from direct exposure, while the remaining five surfaces were directly exposed to the heat. The heating regimen followed the research methodology of Zhenhai Guo [23], employing a heating rate of 10 °C per minute until the target temperature was reached, followed by a constant temperature period of 120 min. The corresponding heating curve is shown in Figure 5.

### 2.4. Mechanical Properties Test of Specimens After High Temperature

**Figure 6:**
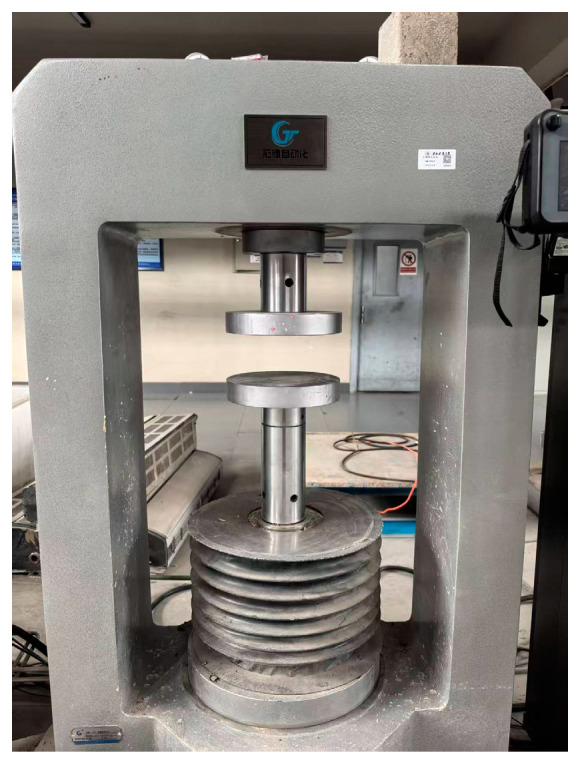
Full-curve testing machine.

Following the high-temperature exposure, the specimens were allowed to cool naturally back to the ambient temperature of 10 °C. Subsequently, they were weighed again, and their post-heating mass was recorded as m_2_. The mechanical properties test was then conducted using a full-curve testing machine, as depicted in Figure 6. The compressive strength value for each concrete cube was directly obtained from the testing machine. The final reported compressive strength for each mix and temperature condition was taken as the average value calculated from the results of three individual specimens.

### 2.5. Non-Contact Deformation Analyses

The surface deformation of a specimen prior to ultimate failure is often minimal and cannot be directly observed with the naked eye. To address this, the present study employs the digital image correlation (DIC) method to conduct a detailed deformation analysis. Compared to traditional physical or mechanical displacement testing techniques, the DIC method offers significant advantages, including operational simplicity, non-destructiveness, full-field measurement capability, non-contact operation, high accuracy, and a high degree of automation. Its core procedural steps involve speckle pattern preparation, sequential image acquisition, the implementation of correlation algorithms, and subsequent strain calculation.

**Figure 7:**
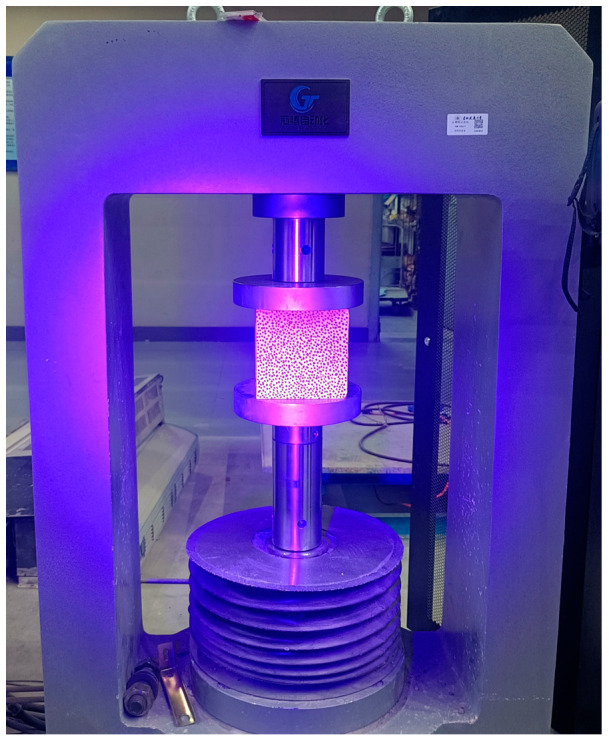
Speckle patterns on test blocks.

**Figure 8:**
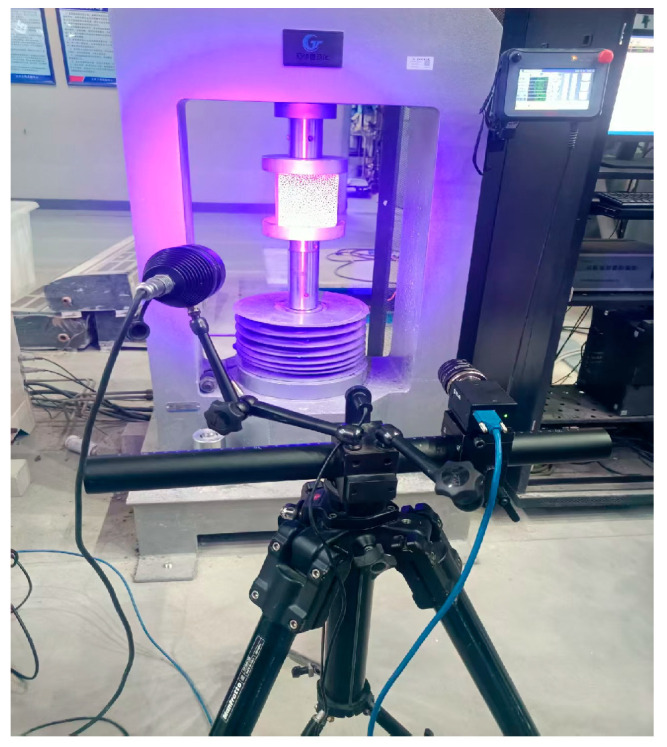
Image acquisition equipment for test blocks.

In this investigation, DIC served as the primary research platform. Specifically, the Thousand Eyes Wolf DIC strain field measurement and analysis system was utilized to collect and process data on the surface displacement field during the uniaxial compression of the high-temperature-exposed concrete cube specimens. This methodology enables the generation of strain maps for the material under different stress states throughout the damage process, thereby facilitating an analysis of the surface damage patterns of the LWC-CB after exposure to varying temperatures. The speckle patterns applied to the test blocks and the corresponding image acquisition equipment setup are shown in Figure 7 and Figure 8, respectively.

## 3. Analysis of Test Results

### 3.1. Visual Inspection

After the specimens were naturally cooled to 10 °C, visual observation revealed distinct characteristics of the LWC-CB specimens under different high-temperature histories, as presented in Table 8.

**Figure 9:**
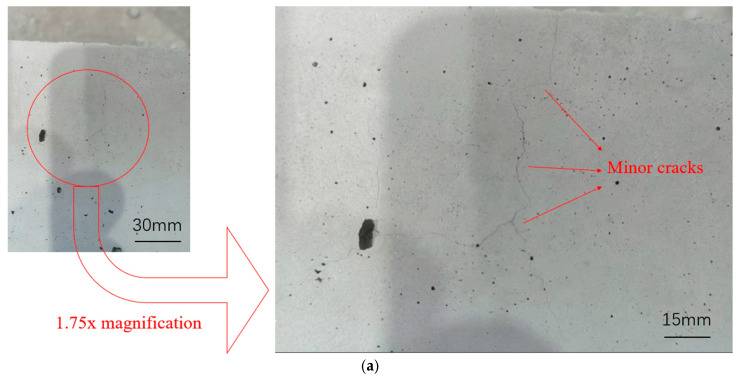

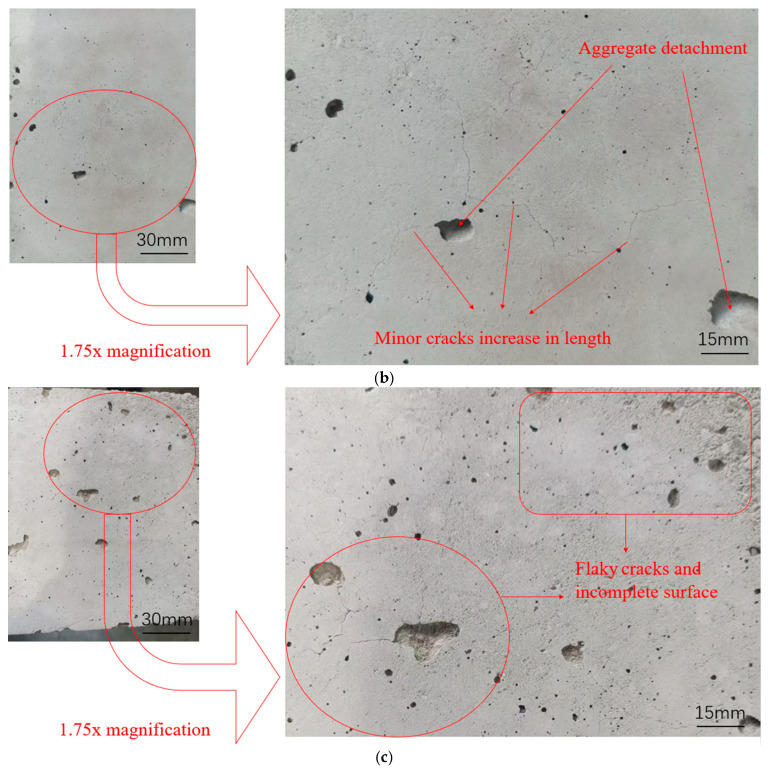
Cracking and surface damage of LWC-CB specimens after different high-temperature effects. (**a**) Surface Phenomena of Specimens at 200 °C. (**b**) Surface Phenomena of Specimens at 400 °C. (**c**) Surface Phenomena of Specimens at 600 °C.

As shown in Figure 9, the test blocks exhibit distinct appearances after high-temperature exposure. At 200 °C, the blocks remain grey. As the temperature rises, their color gradually turns red, with the blocks at 600 °C appearing dark red. With increasing temperature, fine cracks on the top of the blocks become more pronounced, forming flaky damage in some areas at 600 °C. Meanwhile, the surface integrity degrades: the matrix cracks, skin peels off, and CB aggregates dislodge. At 600 °C, surface CB aggregates severely spall, and some blocks even show corner breakage.

### 3.2. Mass Loss Rate

LWC-CB specimen blocks will gradually lose their internal water under the action of high temperature, which includes free water (liquid water present within the concrete interior not involved in chemical reaction) and bound water (water fixed in the cement hydration products through chemical bonding or physical adsorption). Therefore, the mass loss rate of LWC-CB specimens after high temperature can be used to indirectly reflect the damage condition of concrete. The mass loss rate is calculated as Equation (6):

(6) $$m_{L}=\frac{m_{1}-m_{2}}{m_{1}}\times 100\%$$

where

$m_{L}$—Mass loss rate (%);
$m_{1}$—Mass before high temperature (after drying) (kg);
$m_{2}$—Mass after high temperature (kg);

The results of the calculation of the mass loss rate are shown in Table 9.

The mass loss rate after high-temperature heating increases gradually with temperature. Below 200 °C, mass loss in concrete is primarily due to the evaporation of free water inside the concrete caused by high temperature. Above 200 °C, bound water in the cement paste begins to escape. At 400 °C, hydrated calcium silicate and hydrated calcium aluminates in the cement matrix gradually dehydrate, further increasing mass loss. As temperature rises, CH in the cement matrix starts to undergo extensive thermal decomposition, leading to an increase in internal pores of the concrete and a further rise in mass loss [24].

### 3.3. Mechanical Properties of LWC-CB Cubes After High Temperatures

The values of LWC-CB cubic compressive strength with temperature are shown in Table 10. To ensure the reliability and repeatability of the cubic compressive strength results, each temperature group was tested with 3 parallel specimens. According to the table, it can be seen that the compressive strength of the LWC-CB cube shows a decreasing trend with the rise in temperature. At 200 °C, its compressive strength is 94.4% of that at normal temperature. At 400 °C, its compressive strength is 88.3% of that at normal temperature. The compressive strength of the LWC-CB cube decreases sharply at 600 °C, which is only 54.7% of the normal temperature value.

Regression analysis was performed on the cube compressive strength test data of LWC-CB specimens, yielding the following mathematical expression for the relationship between cube compressive strength and temperature (Equation (7a)):

(7a) $$f_{cu}\left(T\right)=({-2\times 10}^{-6}T^{2}+0.0004T+0.9781)f_{cu}(10{}^{\circ}C\leq T\leq 600{}^{\circ}C)$$

Wenting Jiang [25] investigated the application of sintered sludge aggregates as a full replacement for natural aggregates in concrete. The mathematical expression between the cubic compressive strength and temperature is obtained as follows (Equation (7b)):

(7b) $$\left\{\begin{array}{c} f_{cu}\;\left(T\right)=\left(1.004-0.198T\times 10-3\right)f_{cu}\;T\;\leq 200\;{}^{\circ}C\\ \;f_{cu}\;\left(T\right)=\left[\begin{array}{c} 0.961-0.173\;\times \;\left(T-200\right)\times 10^{-3}\\ -1.101\;\times \;{\left(T-200\right)}^{2}\;\times 10^{-6} \end{array}\right]f_{cu}\;T\;>\;200\;{}^{\circ}C \end{array}\right.$$

In the literature [14], Zhenhai Guo presents the fitting equation for the variation in the cubic compressive strength of natural aggregate concrete with temperature (Equation (7c)):

(7c) $$f_{cu}\left(T\right)=\frac{f_{cu}}{1+{2.4(T-20)}^{6}\times 10^{-17}}$$

where

T: Specimen heating temperature (°C);
$f_{cu}$: Cubic compressive strength of concrete at room temperature (MPa);
$f_{cu}\left(T\right)$: Cubic compressive strength of concrete after high-temperature heating (MPa).

**Figure 10:**
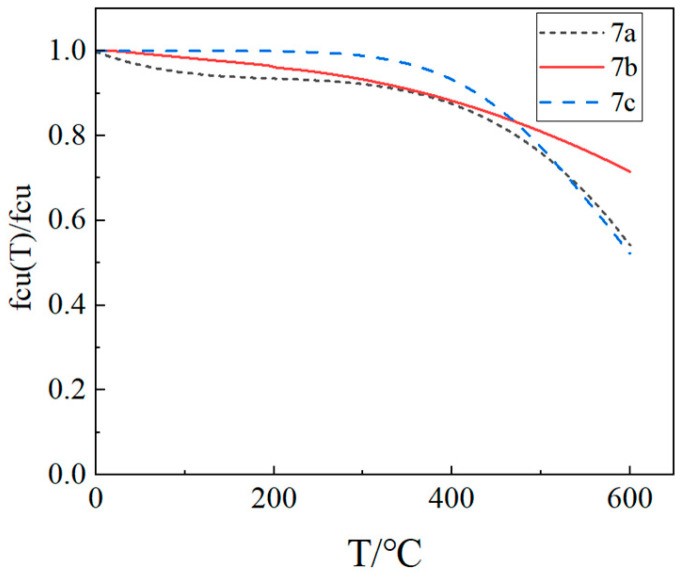
Comparison of residual strength curves for Equations (7a)–(7c).

The comparison curve of the above three formulas is shown in Figure 10:

As illustrated in Figure 10, the cubic compressive strength ratio curve of LWC-CB specimens demonstrates a trend analogous to that of natural aggregate concrete, with a slight reduction relative to natural aggregate concrete at 500 °C. Up to 450 °C, the residual compressive strength ratio of natural aggregate concrete remains marginally higher than that of sintered aggregate concrete and CB aggregate concrete; however, beyond 450 °C, the residual compressive strength ratio of sintered aggregate concrete begins to distinctly exceed that of natural aggregate concrete and CB aggregate concrete.

### 3.4. Destruction Mechanism of LWC-CB Specimen After 10 °C and High Temperature

At 10 °C, the strength of CB aggregates is lower than that of the mortar, and consequently, the strength of the CB aggregates effectively determines the strength of the LWC-CB. Micro-strain in the specimen during compression was observed using DIC equipment. Table 10 below presents the longitudinal (uniaxial compression direction) and transverse (direction perpendicular to uniaxial compression) strain contour plots of 10 °C specimens under different stress levels, obtained via DIC. Figure 10 illustrates the post-damage morphology of LWC-CB after the 10 °C compression test.

10(a), 10(b), 10(c), 10(d), 10(a′), 10(b′), 10(c′), 10(d′): “10” indicates a temperature of 10 °C. The letters in parentheses (a, b, c, d, a′, b′, c′, d′) identify different test conditions, specimens or test stages. They are used to distinguish strain test results under different conditions at 10 °C.

When observing the above figures at 10 °C during the initial loading stage, the transverse strain field ε_x_ remains relatively stable, with strain values at the top and bottom edges of the specimen being higher than those at the center. They are 267.6 με and −203.1 με, respectively. This is consistent with the findings of studies by Jinna Shi [26] and Grzegorz Ludwik Golewski [27]. Meanwhile, stress concentration emerges in specific regions of the top and bottom edges, as depicted in 10(a) within Table 10. For the longitudinal strain ε_y_, the specimen is under compression at this stage, and internal gaps within the specimen are closed by pressure. The specimen gradually compresses, with the maximum compression value appearing at the top and bottom of the specimen. The strains are 367.6 με and −381.8 με. As shown in 10(a′) within Table 11.

As the load continues to increase to 0.3 *f*_cT_, both longitudinal and transverse strains increase. In the transverse strain contour plot, obvious stress concentration appears in specific regions of the top and bottom edges, corresponding to the longitudinal strain contour plot. It concentrates on the lower left corner of the test block, with the maximum values of longitudinal strain and transverse strain being −1360.4 με and 881.0 με, respectively. In the longitudinal strain contour plot, the compression zone of longitudinal stress spreads from the edge regions to the middle. At 0.8 *f*_cT_, in the transverse strain field ε_x_, stress concentration zones at the top and bottom edges spread to the center along vulnerable regions. The longitudinal stress compression region not only extends inward but also spreads laterally, with the maximum compression of the longitudinal strain region appearing in the stress concentration zones corresponding to ε_y_.

At peak stress *f*_cT_, since the tensile strength of concrete itself is much lower than its compressive strength, with increasing load, transverse deformation occurs due to the Poisson effect. This deformation is constrained by the surrounding concrete, generating transverse tensile stresses. When this tensile stress exceeds the tensile strength of concrete, the specimen develops cracks. As shown in the figures, the transverse strain increases sharply to 7067.3 με, corresponding to a longitudinal strain region of −1920.4 με. The strain concentration zones in the transverse strain contour plot form through-cracks across the specimen as the load increases. The crack initiation process is consistent with that captured by DIC equipment in studies by Jinna Shi [26] and Grzegorz Ludwik Golewski [27].

**Figure 11:**
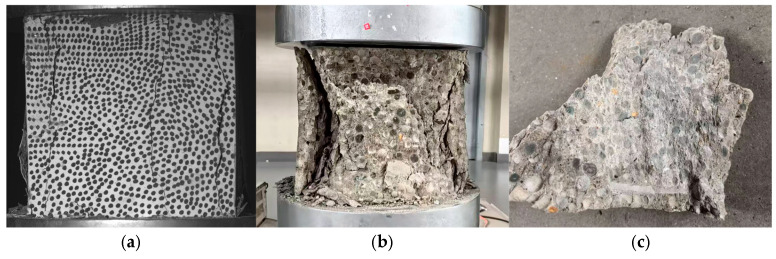
Morphology of LWC-CB after damage in compressive test at 10 °C. (**a**) Crack maps of test blocks recorded by DIC. (**b**) The final damage morphology of the specimen. (**c**) Fragments dislodged during the test.

Subsequently, due to excessive deformation, the DIC system was disengaged, and continuous recording was performed using a camera. As the load increased, longitudinal cracks propagated progressively, as shown in Figure 11a, accompanied by fragment dislodgment. The final damage morphology of the specimen, depicted in Figure 11b, exhibits a quadrangular cone structure with positive-inversion connection. Observing the test block’s final damage morphology and fragment shedding in Figure 11c, it is evident that the aggregates were neatly sheared off.

In natural aggregate concrete, the ITZ between coarse aggregates and mortar serves as the weak link, so interfacial damage typically dominates during cube compression tests. At 10 °C, the microstructure of the interface zone is relatively dense. When the temperature reaches 200~300 °C, CH begins to dehydrate, crystal lattices undergo severe distortion, pore channels increase, and numerous interfacial cracks form. These processes severely weaken interfacial bonding, causing a sharp decline in load-bearing capacity. After 400 °C, this degradation gradually intensifies with increasing temperature [28,29].

For LWC-CB specimens, the micro-strain during compression was observed using DIC equipment. The longitudinal and transverse strains of the specimens under uniaxial compression at different stress levels after exposure to various high temperatures are presented in Table 12.

When observing specimens at 200 °C, 400 °C, and 600 °C, partial peeling of the surface occurs after high-temperature loading due to pressure, resulting in white areas in the strain contour plots. This effect becomes more pronounced with increasing temperature. For transverse and longitudinal strain contour plots at 0.3 *f*_cT_, 0.8 *f*_cT_, and *f*_cT_, compared with the 10 °C concrete, the transverse strain contour plots of high-temperature-exposed concrete exhibit stress concentration in edge regions at the initial stage. Moreover, the test blocks exhibit relatively large strain values at the initial loading stage. For instance, under the conditions of 200 °C, 400 °C, and 600 °C, the maximum transverse strain values are generally more than twice those at 10 °C. At the same stress level, the stress concentration area is larger at higher temperatures, with a clear trend of expansion as temperature increases. At 0.3 *f*_cT_, 0.8 *f*_cT_, and *f*_cT_, the maximum transverse strain values under 200 °C, 400 °C, and 600 °C are 5.4 times, 6.1 times, and 9 times those at 10 °C, respectively.

In the longitudinal strain contour plots, longitudinal compression strain propagates rapidly across the entire plane. Although the maximum longitudinal compression strain appears in the region corresponding to the transverse strain concentration area, the range of this maximum strain region expands with increasing stress ratio and temperature. After high-temperature exposure, the specimen becomes loose, with severe damage to particle connections and structural integrity. The internal structure cannot co-load efficiently after loading, leading to delayed force transfer. This results in a mismatch between transverse and longitudinal strain concentration areas, causing premature failure of the transverse strain concentration regions before full propagation.

Jinna Shi [26] investigated the deformation properties of ordinary concrete after high temperatures. Ordinary concrete also has the following characteristics. First, the higher the temperature, the larger the stress concentration area. It tends to expand as temperature rises. Second, after high-temperature exposure, the test blocks become loose. There is a mismatch between transverse and longitudinal strain concentration areas. However, LWC-CB shows these two characteristics more obviously than ordinary concrete. This also explains why the compressive strength of LWC-CB is lower than that of ordinary concrete after 500 °C.

**Figure 12:**
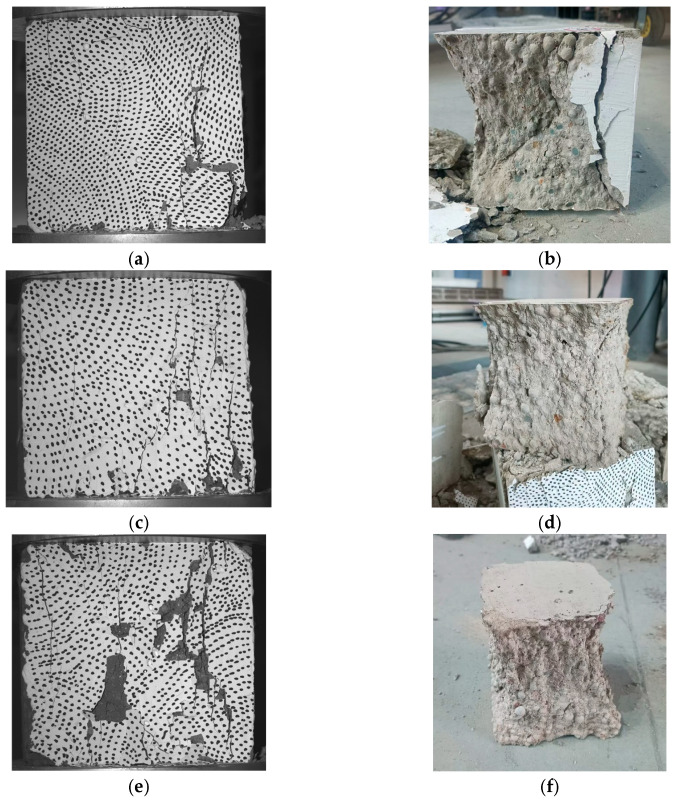
Damage morphology of LWC-CB after compressive tests at different temperatures. (**a**) Crack maps of test blocks recorded at 200 °C. (**b**) The final damage morphology at 200 °C. (**c**) Crack maps of test blocks recorded at 400 °C. (**d**) The final damage morphology at 400 °C. (**e**) Crack maps of test blocks recorded at 600 °C. (**f**) The final damage morphology at 600 °C.

After the DIC system was disengaged, crack propagation was recorded using a camera, as shown in Figure 12a,c,e. The final damage morphology of the specimens, depicted in Figure 12b for each temperature, also exhibits a positive inversion connecting the tetragonal cone. Observing the specimens’ final damage morphology, it is evident that with increasing temperature, CB aggregates are not gradually sheared; instead, internal cracks within the specimens progressively increase, and the mortar structure becomes loose.

For the specimen heated to 200 °C, the CB aggregates in the middle of the damage surface are crushed, while the edge regions of CB aggregates detach and crack from the surrounding mortar, indicating that the bonding strength between CB aggregates and mortar begins to weaken due to high temperature. At 400 °C, CB aggregates are basically no longer crushed, but mortar cracks widen and extend to through-cracks. At this temperature, CH within the mortar starts to dehydrate and decompose, leading to significant reduction in CH quantity, crystal defects, loose structure, pore cracks, and interfacial cracks with CB aggregates, which decreases the bonding strength between CB aggregates and mortar.

As the temperature continues to rise, these structural changes in the mortar gradually intensify, causing continuous strength degradation and severe weakening of interfacial bonding force. Consequently, more interfacial cracking damage occurs between CB aggregates and mortar, as shown in Figure 12f damage morphology, where most of the mortar is crushed into powder. At this stage, the strength-determining factors of LWC-CB are no longer solely CB aggregates but also depend on mortar strength and interfacial adhesion.

### 3.5. Analysis and Discussion

The compressive strength of LWC-CB exhibits a characteristic stepwise degradation with increasing temperature. While the strength retained 94.4% and 88.3% of its ambient value at 200 °C and 400 °C, respectively, it underwent a sharp decline to 54.7% at 600 °C. Compared with ordinary concrete, before 500 °C, the strength retention effect of LWC-CB is 6% better than that of ordinary concrete; beyond 500 °C, the strength retention effects of the two types of concrete tend to be consistent.

At ambient temperature (10 °C), LWC-CB specimens exhibited a characteristic shear failure mode under compression. The failure surface revealed neatly fractured ceramsite (CB) aggregates, indicating that these aggregates act as the primary weak link within the composite system.

Exposure to high temperature significantly altered both the mechanical properties and failure mechanisms. The compressive strength demonstrated a progressive degradation, with the residual strength at 600 °C measured at only 54.7% of the ambient value, a trend consistent with that observed in ordinary concrete. This performance loss is attributed to microstructural changes. Thermally induced dehydration and decomposition of portlandite (CH) within the cement mortar generate an internal microcrack network, reducing its intrinsic strength. Concurrently, the mismatch in thermal expansion coefficients between the mortar and the CB aggregates induces additional stresses at the Interfacial Transition Zone (ITZ), exacerbating interfacial bond degradation. Consequently, the post-high-temperature damage path primarily localizes within the mortar matrix and the ITZ. The residual compressive strength is therefore governed by the synergistic deterioration of the mortar and the integrity of the interfacial bonds.

## 4. Numerical Simulation

### 4.1. Concrete Model

**Figure 13:**
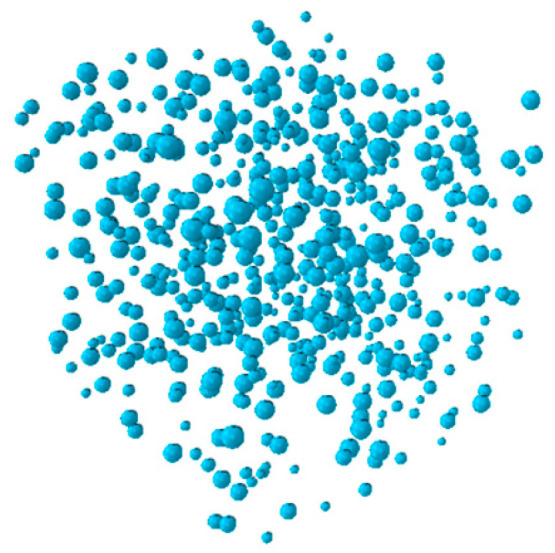
CB Aggregate.

To further investigate the micromechanical behavior identified experimentally, a three-dimensional mesoscale model was developed using ABAQUS. This approach, well-established in concrete research [30,31], explicitly represents LWC-CB as a three-phase composite comprising CB aggregates, mortar, and the ITZ. The model was designed specifically to analyze stress evolution and damage initiation consistent with the observed physical phenomena. In this study, the spatial distribution of aggregates was determined using Monte Carlo random numbers, and a three-dimensional mesoscale model was established based on the Fuller grading curve [32]. The random generation and placement of aggregates are implemented using Python 3.12 programming language, with three rules to be followed during the aggregate placement process: (1) the geometric positions of aggregates are randomly distributed within the placement area; (2) a certain distance is maintained between aggregates and the model boundary to serve as the concrete cover; (3) aggregates placed later must not intersect or overlap with those placed earlier. The three-dimensional aggregate distribution model is shown in Figure 13.

**Figure 14:**
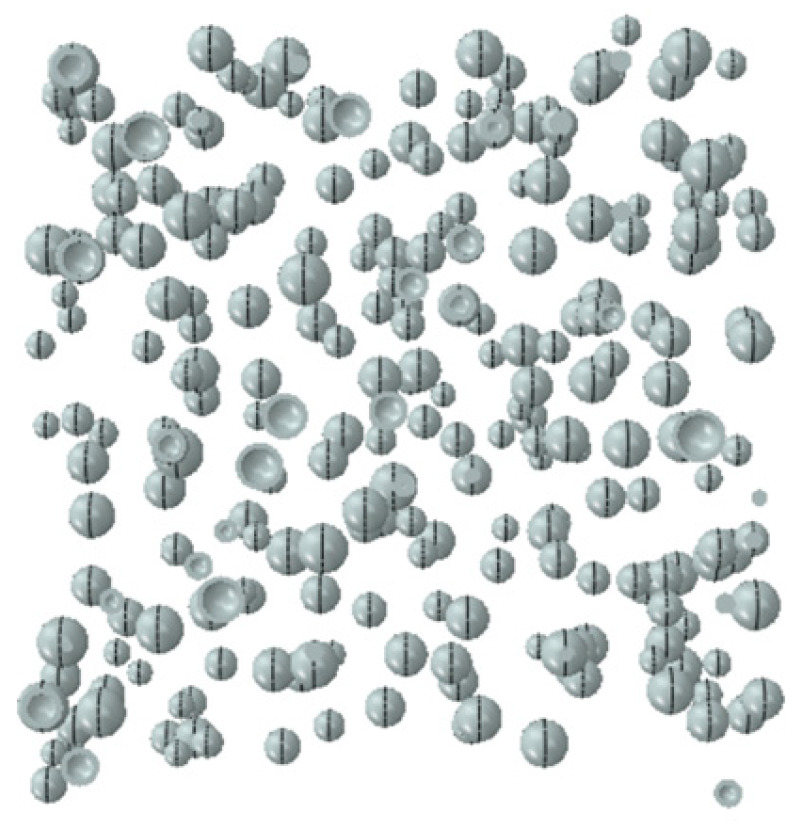
Interfacial transition zone (ITZ).

**Figure 15:**
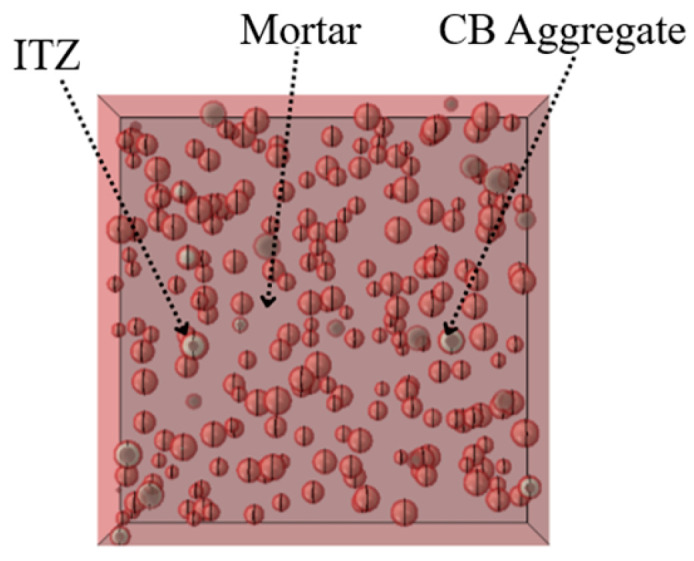
General model after assembly.

**Figure 16:**
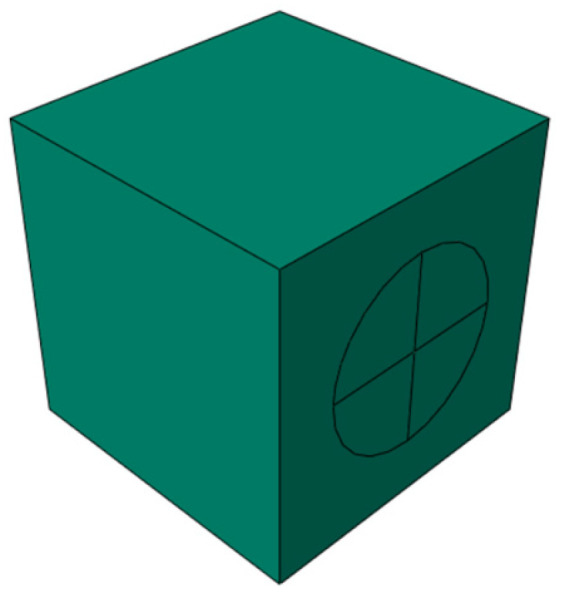
The model after different temperature exposures.

Referring to the numerical simulation results in the literature [33,34], when the thickness of the ITZ ranges from 0.5 to 2 mm, it primarily affects only the descending branch of the concrete stress–strain curve. Therefore, the ITZ thickness in this study was set to 1 mm, as shown in Figure 14, with the overall model depicted in Figure 15. Given the high computational cost and the limited existing research on the high-temperature performance of LWC-CB, this paper performs random aggregate modeling specifically for the 10 °C LWC-CB case, while adopting a homogeneous modeling approach for the different temperature scenarios, as shown in Figure 16.

### 4.2. Constitutive Model of 3D Mesoscale Model at 10 °C

The LWC-CB three-dimensional mesoscale model is a mesoscopic finite element model, for which macroscopic constitutive relations are no longer directly applicable. However, research on the constitutive relations of mortar and CB aggregates remains limited, particularly for CB aggregates, whose constitutive parameters are nearly impossible to measure directly through experiments. Previous studies suggest that the mechanical behavior of the mortar matrix is analogous to that of conventional concrete. Therefore, this paper adopts the plastic damage constitutive model of LC25 concrete to characterize the mortar phase. The mechanical properties of the LC25-strength LWC-CB component were calculated using the following Equations (8)–(13) [35,36].

Axial compressive strength *f*_c_:

(8) $$f_{c}=0.880f_{cu}$$

Peak strain in axial compression $\varepsilon_{c}$:

(9) $$\varepsilon_{c}=730f_{cu}^{1/3}\times 10^{-6}$$

Axial tensile strength f_t_:

(10) $$f_{t}=f_{cu}/15$$

Peak strain in axial tension $\varepsilon_{t}$:

(11) $$\varepsilon_{t}=20f_{cu}^{2/3}\times 10^{-6}$$

Modulus of elasticity $E_{c}$:

(12) $$E_{c}=2.020\rho f_{cu}^{0.5}$$

where

*f*_cu_—Cubic specimen compressive strength (MPa);
ρ—Apparent density of concrete (kg/m^3^).

The LWC-CB stress–strain full-curve equation is adopted as follows:

(13) $$y=\left\{\begin{array}{c} \frac{c_{n}+\left(d_{n}-1\right)x^{2}}{1+\left(c_{n}-2\right){x+d_{n}x}^{2}},x\leq 1\\ \frac{x}{b_{n}{\left(x-1\right)}^{2}+x},x\geq 1 \end{array}\right.$$

**Figure 17:**
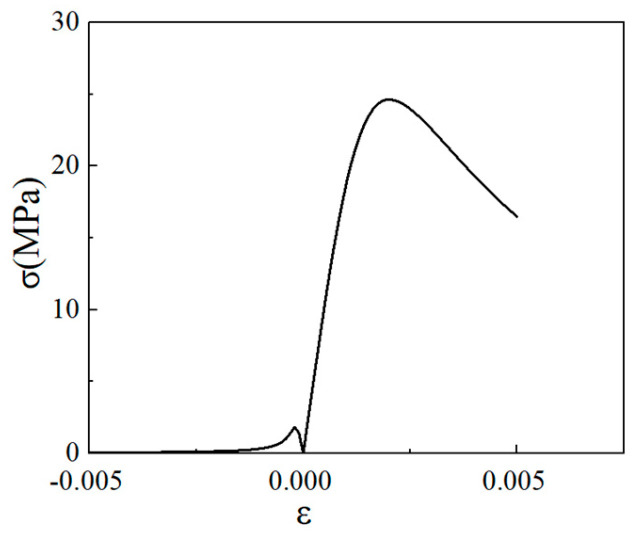
Full-curve equation of stress–strain of LWC-CB at 10 °C.

The plotted curve is shown in Figure 17.

Following the research by [37], the strength of the ITZ is lower than that of the mortar. Due to the lack of relevant experimental data, the ITZ strength is generally determined by multiplying the mortar strength by a reduction factor. In this study, the plastic damage model of concrete is adopted to characterize the ITZ. According to relevant literature [38], defining the ITZ strength as approximately 0.785 times the mortar strength is appropriate. Given the low strength of CB aggregates, the damage morphology of LWC-CB concrete differs from that of ordinary concrete, with cracks tending to initiate from the aggregates first. To describe this phenomenon, the plastic damage constitutive model is also applied to characterize the mechanical behavior of the aggregates. Since CB aggregates belong to complex-phase materials, directly using their nominal strength is inaccurate. Instead, their strength is mostly determined via the cylinder compression strength calibration method. Through extensive experimental studies, Liu et al. [39] established an effective elastic modulus relationship equation for ceramic aggregates, as shown in Equation (14).

(14) $$E_{ea}=0.0106\rho_{a}^{2}$$

where

*E*_ea_—Modulus of elasticity of ceramic aggregates (MPa);
*ρ*_a_—Apparent density of aggregates (kg/m^3^).

### 4.3. The Constitutive Relationship of LWC-CB After Different Temperatures

The plastic damage model is applied to LWC-CB after exposure to different temperatures. Given the limited research on LWC-CB after high-temperature exposure, a formula derived from previous studies on lightweight aggregate concrete after different temperatures is adopted here for subsequent simulations, as it closely approximates the behavior of LWC-CB [40]. (Equations (15)–(18))

(1) Compressive and tensile strength of concrete after high temperature:

(15) $$\frac{f_{c,T}}{f_{c}}={-\left(\frac{T}{1000}\right)}^{2}+3\left(\frac{T}{1000}\right)+0.02\left(25\;{}^{\circ}C<T\leq 600\;{}^{\circ}C\right)$$

where

$f_{c}$—Axial compressive strength of lightweight aggregate concrete at room temperature (MPa);
$f_{c,T}$—Axial compressive strength of lightweight aggregate concrete at high temperature (MPa).

(16) $$\frac{f_{t,T}}{f_{t}}\left\{\begin{array}{c} 0.58\left(1-\frac{T}{300}\right)+0.42\;\left(0\;{}^{\circ}C\leq T\leq 300\;{}^{\circ}C\right)\\ 0.42\left(1.6-\frac{T}{300}\right)\;\left(300\;{}^{\circ}C<T\leq 800\;{}^{\circ}C\right)\\ 0\;\left(T>800\;{}^{\circ}C\right) \end{array}\right.$$

where

$f_{t}$—Tensile strength of concrete at room temperature (MPa);
$f_{t,T}$—Tensile strength of concrete at high temperature (MPa).

(2) Modulus of elasticity

(17) $$\frac{E_{cT}}{E_{c}}=1.026-1.0\left(\frac{T}{1000}\right)-0.6\left(\frac{T}{1000}\right)\left(25\;{}^{\circ}C\leq T\leq 600\;{}^{\circ}C\right)$$

where

$E_{c}$—Modulus of elasticity of lightweight aggregate concrete at room temperature (MPa);
$E_{cT}$—Modulus of elasticity of lightweight aggregate concrete after high temperature (MPa).

(3) Stress–strain relationship of concrete under compression after high temperature:

(18) $$\frac{\sigma_{c}}{\sigma_{c}\left(T\right)}=\left\{\begin{array}{c} \alpha \left(\frac{\varepsilon_{0}}{\varepsilon_{0r}\left(T\right)}\right)+\left(5-4\alpha \right){\left(\frac{\varepsilon_{0}}{\varepsilon_{0r}\left(T\right)}\right)}^{4}+\left(3\alpha -4\right){\left(\frac{\varepsilon_{0}}{\varepsilon_{0r}\left(T\right)}\right)}^{5}\;\left(0\leq \left(\frac{\varepsilon_{0}}{\varepsilon_{0r}\left(T\right)}\right)\leq 1\right)\\ \frac{\frac{\varepsilon_{0}}{\varepsilon_{0r}\left(T\right)}}{\beta \left(\frac{\varepsilon_{0}}{\varepsilon_{0r}\left(T\right)}-1\right)+\frac{\varepsilon_{0}}{\varepsilon_{0r}\left(T\right)}}\;\left(\frac{\varepsilon_{0}}{\varepsilon_{0r}\left(T\right)}>1\right) \end{array}\right.$$

$$\left\{\begin{array}{c} \alpha =1.4268-0.0016T\\ \beta =5.3606+0.0078T-0.000009T^{2} \end{array}\right.$$

where

$\sigma_{c}$—Compressive stress of lightweight aggregate concrete (MPa);
$\sigma_{c}\left(T\right)$—Peak compressive stress of lightweight aggregate concrete after exposure to temperature T (MPa);
$\varepsilon_{0}$—Compressive strain of lightweight aggregate concrete;
$\varepsilon_{0r}\left(T\right)$—Peak compressive strain of lightweight aggregate concrete after exposure totemperature T;
α,β—Shape parameters controlling the ascending and descending branches of the stress–strain curve, which can be determined by linear interpolation using the relevant formulas.

**Figure 18:**
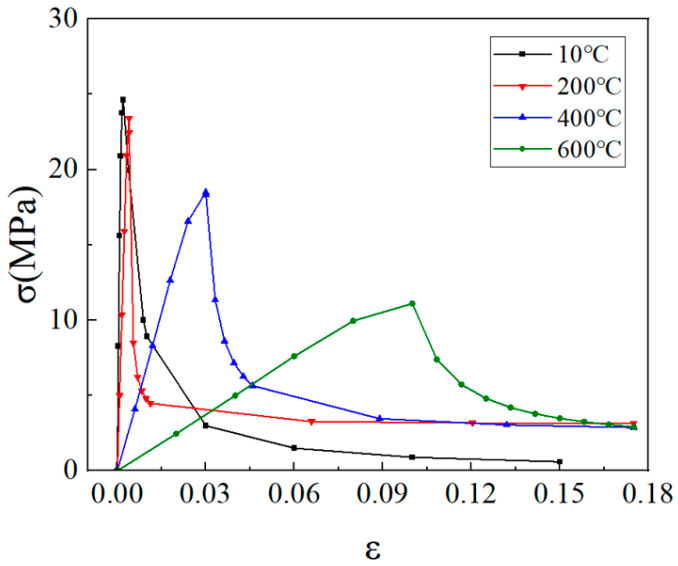
Compressive stress–strain relationship of LWC-CB after high temperature.

Based on the above literature, the stress–strain relationship after fire of LWC-CB is plotted as shown in Figure 18.

(4) Tensile stress–strain relationship after high temperature

**Figure 19:**
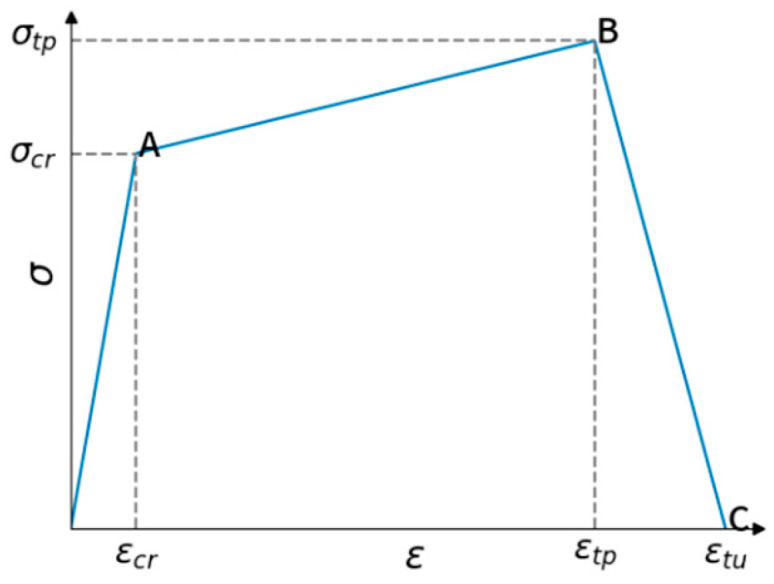
Tensile stress–strain relationship of LWC-CB after high temperature.

Regarding the tensile behavior of LWC-CB, this study adopts the three-linear constitutive relationship proposed by Han et al. [41], which considers the elastic stage, strain hardening stage, and softening stage after the peak stress. The constitutive relationship is shown in Figure 19. In the figure, σ_cr_ and ε_cr_ are the tensile cracking stress and strain, σ_tp_ and ε_tp_ are the peak tensile stress and strain, and ε_tu_ is the tensile strain at complete failure. The influence of parameters and temperature refers to the mechanical property research on lightweight aggregate concrete under high temperature by Haiyang Shen [42] and Equations (16) and (17). The above various parameters are summarized in Table 13.

The remaining parameters are taken as a density of 1.9 × 10^−9^ g/mm^3^, a dilation angle of 30°, an eccentricity of 0.1, a stress ratio of 1.16 and a viscosity coefficient of 0.05.

### 4.4. Meshing

#### 4.4.1. Meshing of 3D Mesoscale Model

**Figure 20:**
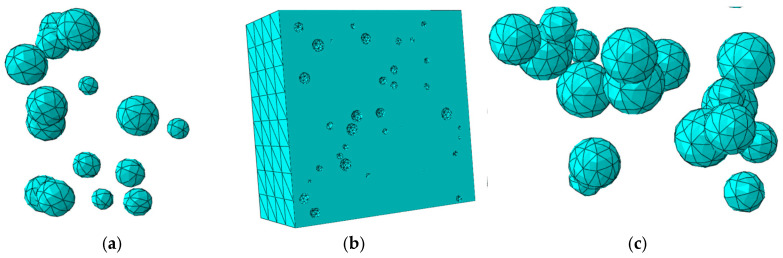
Meshing of 3D mesoscale model: (**a**) CB aggregate; (**b**) mortar; (**c**) ITZ.

Considering the high computational cost, the mesh size for CB aggregates and the ITZ was controlled at 1 mm during model meshing. The mesh size at the interface between the mortar and ITZ was also set to 1 mm, with appropriate mesh coarsening in regions away from the interface, as shown in Figure 20.

#### 4.4.2. Mesh Delineation of Concrete Model After Different Temperatures

**Figure 21:**
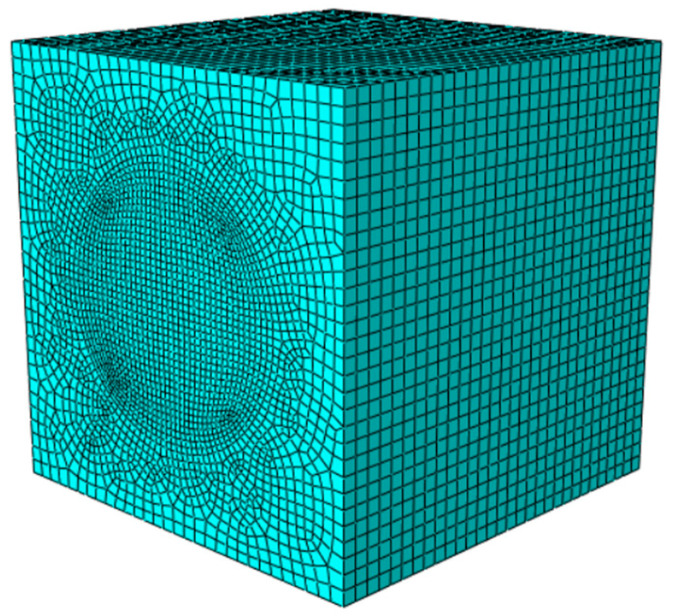
Mesh of concrete specimen after high temperature.

For the mesh delineation of the concrete model after different temperatures, the global mesh size was set to 5 mm. To better observe the specimen damage and simulate internal defect propagation, the internal mesh was locally refined, as shown in Figure 21. The comparison of computational efficiency is presented in Table 14 and Table 15.

### 4.5. Modeling Feasibility Analysis

**Figure 22:**
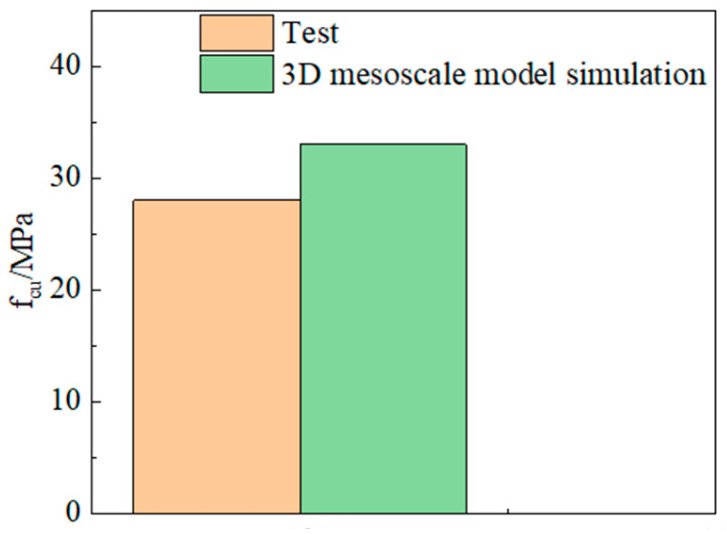
Comparison between the 3D mesoscale model simulation and the test result at room temperature.

**Figure 23:**
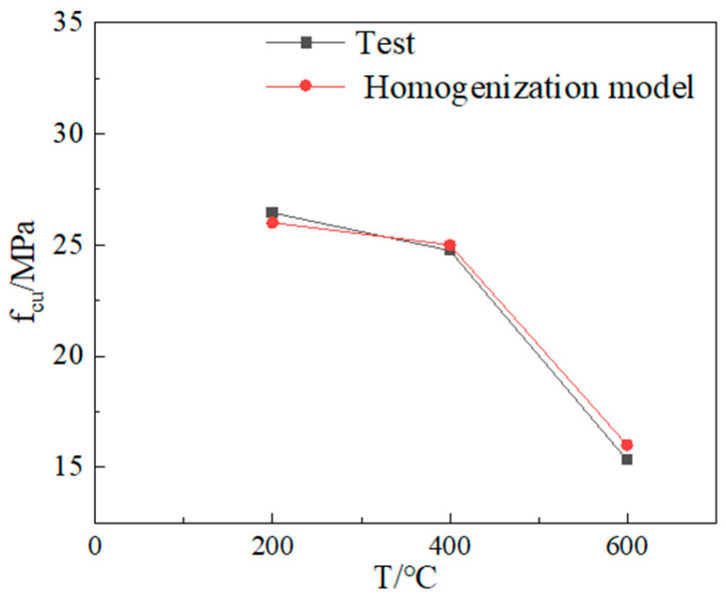
Comparison between homogenization model simulation and test results after exposure to different temperatures.

The comparison between simulation and test results is shown in Figure 22 and Figure 23.

At room temperature, the compressive strength from the 3D mesoscale model simulation differs from the experimental value by 15%. After exposure to different temperatures, the maximum difference between the compressive strength from the concrete model simulation and the experimental value is 3%. Demonstrates that the test data align closely with the simulated values, indicating excellent agreement between the two. Therefore, the three-dimensional mesoscale model and the concrete models after different temperatures established in this study can be reliably used to analyze the damage mechanism of LWC-CB.

### 4.6. Analysis of Finite Element Simulation Results for Three-Dimensional Mesoscale Model

**Figure 24:**
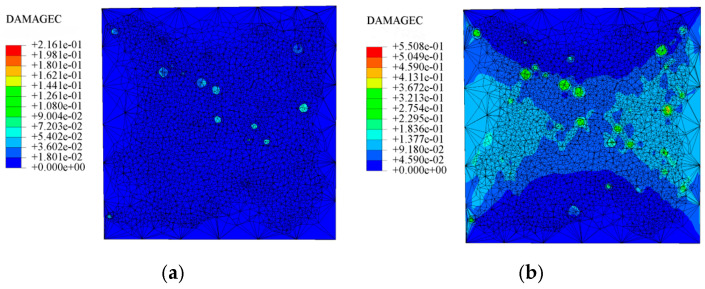

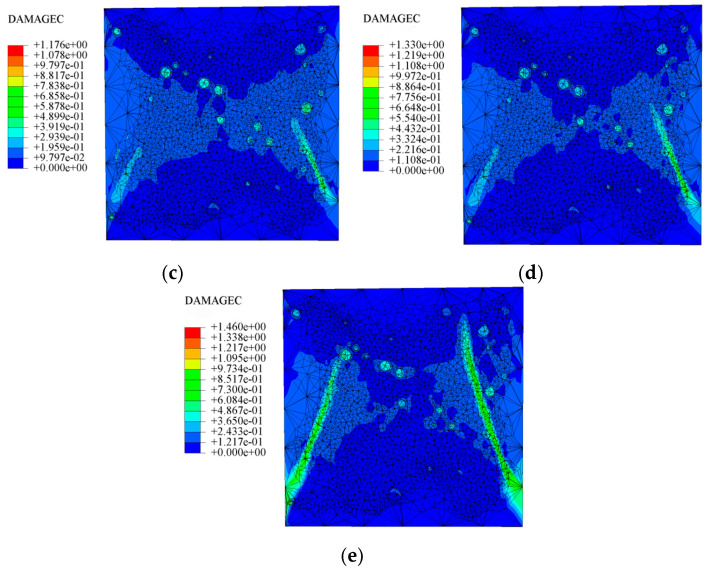
Compressive damage development diagram of LWC-CB model: (**a**) elastic stage; (**b**) damage accumulation stage; (**c**) crack initiation stage; (**d**) crack propagation stage; (**e**) oblique through crack.

Figure 24 shows the compression damage contour of the internal profile of the LWC-CB model under compressive loading from the simulation, which reflects the compression damage mode of the LWC-CB and indicates that the concrete element has been completely destroyed when the damage factor reaches more than 0.9. At the beginning of the simulation, the LWC-CB model is in the elastic stage, as shown in Figure 24a. With the gradual application of load, the internal damage of the concrete starts to accumulate at the CB aggregates, where the CB aggregate portion first undergoes damage and produces tiny cracks, as shown in Figure 24b,c. Subsequently, the cracks continue to develop along the periphery and interior of the CB aggregates, ultimately forming an oblique penetration crack.

The damage contours in Figure 24d,e show that the compression damage of LWC-CB is mainly manifested as shear damage, with the resulting cracks propagating obliquely through the ceramic aggregate at an angle of about 45° to the horizontal plane and eventually traversing the concrete. As the phase with the lowest strength among the three-phase materials of this ceramic concrete, the CB aggregate is always damaged first when compression occurs. The damage pattern obtained from the simulation is consistent with the test results of the LWC-CB specimen at 10 °C, which further verifies the validity of the model.

### 4.7. Analysis of Model Results After Different Temperatures

**Figure 25:**
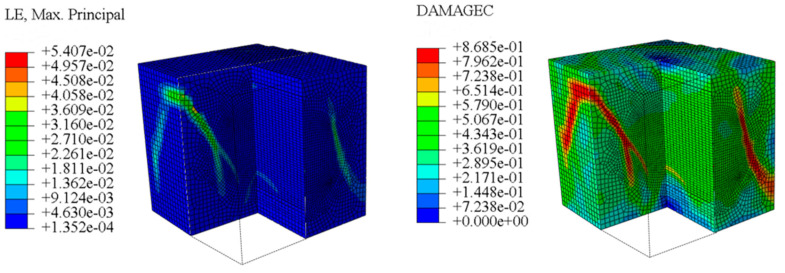
Strain and damage contour of the 10 °C model.

**Figure 26:**
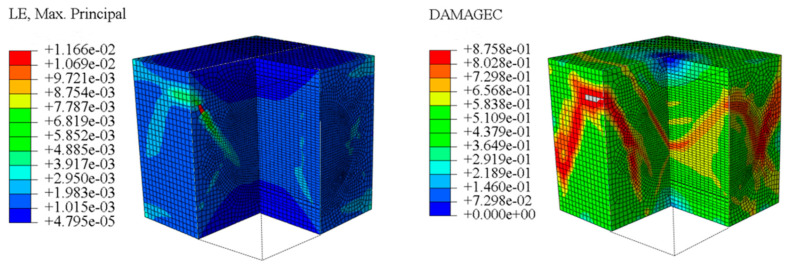
Strain and damage contour of the 200 °C model.

**Figure 27:**
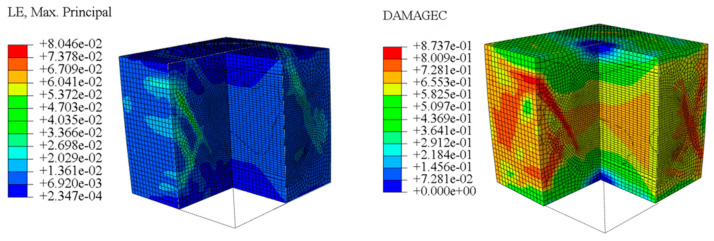
Strain and damage contour of the 400 °C model.

**Figure 28:**
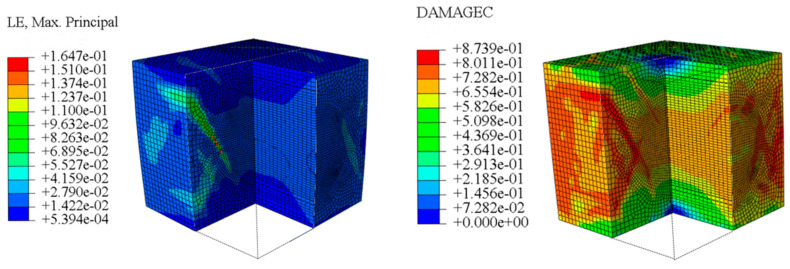
Strain and damage contours of the 600 °C model.

The concrete was simulated using ABAQUS after different temperature exposures. The strain contours and damage contours of the internal profile of the model at the peak stress stage are shown in Figure 25, Figure 26, Figure 27 and Figure 28.

Observation of the LWC-CB model’s strain and damage at 10 °C indicates that most regions exhibit relatively uniform strain and damage distributions, with slightly higher values in parts of the diagonal region. As loading progresses, strain and damage in this region continue to evolve, forming a diagonal penetrating crack that ultimately induces shear failure of the specimen. The simulated damage pattern is largely consistent with both the three-dimensional mesoscale model and experimental results, validating the model’s reliability.

By comparing the cloud diagrams at various temperatures, the following research results were obtained:

At 10 °C, the strain of the LWC-CB model is relatively low and focused in one specific area. When the temperature increases, the color range of the strain cloud diagram shifts to indicate higher strain values. The internal strain of the model becomes significantly larger, and the area characterized by high strain keeps expanding.

At 10 °C, the LWC-CB model exhibits little damage, confined to a small area. As temperature rises, the material’s internal microstructure sustains more damage. In the damage map, the red area (indicating high damage) grows substantially. When the temperature reaches 600 °C, the concrete’s damage increases sharply, demonstrating the material’s significant degradation under high heat.

Based on experimental and numerical simulation methods, the mechanical behavior and damage mechanism of LWC-CB at 10 °C and at different high temperatures are revealed and reproduced. The simulation results form good cross-validation with the experimental data, providing a reliable numerical basis for revealing the performance evolution law of LWC-CB concrete in high-temperature environments.

## 5. Conclusions

In this study, LWC-CB was prepared by fully replacing natural coarse aggregate with fly ash ceramsite (CB) aggregate produced through the cold-bonding method. This study systematically investigated the post-fire behavior of lightweight ceramsite concrete (LWC-CB) through an integrated experimental and numerical approach. The principal findings are as follows:

The compressive strength of LWC-CB exhibits a characteristic stepwise degradation with increasing temperature. While the strength retained 94.4% and 88.3% of its ambient value at 200 °C and 400 °C, respectively, it underwent a sharp decline to 54.7% at 600 °C. Compared with ordinary concrete, before 500 °C, the strength retention effect of LWC-CB is 6% better than that of ordinary concrete; beyond 500 °C, the strength retention effects of the two types of concrete tend to be consistent.

At ambient temperature (10 °C), failure is governed by the shear fracture of the low-strength ceramsite (CB) aggregates, identifying them as the primary weak link. After high-temperature exposure, damage is exacerbated by two synergistic factors: the formation of a microcrack network from mortar dehydration and additional damage at the Interfacial Transition Zone (ITZ) due to thermal expansion mismatch. The post-fire residual strength is thus predominantly controlled by the extent of mortar deterioration and ITZ debonding.

Mesoscale numerical simulations corroborated the experimental findings. The model successfully replicated the stress concentration and initial cracking within CB aggregates at ambient temperature. Furthermore, it quantified the progressive damage evolution from 200 °C to 600 °C, revealing a transition from localized strain to full-field distribution, with the damaged zone area expanding by approximately four times and peak strain increasing 2~3 fold at 600 °C. At room temperature, the compressive strength obtained from the simulation of the 3D mesoscale model differs from the experimental value by 15%. After exposure to different temperatures, the maximum deviation between the compressive strength from the concrete model simulation and the experimental value is 3%. The numerical simulation results show a high degree of agreement with the experimental findings.

The findings of this study have practical applications in structural engineering and sustainable development. They provide mechanical parameter support for assessing and repairing LWC-CB structures after fire and realise the utilisation of industrial solid waste by using cold-bonded (CB) fly ash aggregates. This offers a technical approach to achieving China’s “dual carbon” goals in the construction industry. However, the study has certain limitations. The test temperature range only covers 10–600 °C and does not involve high-temperature scenarios, so it cannot fully reflect the impact of high temperatures that may occur in fires on material properties. The microstructure of the material under high temperatures has not yet been analyzed. In the future, the research group will simulate environments above 600 °C and simultaneously conduct more in-depth research into the deterioration of LWC-CB’s mechanical properties at a microscopic level.

## Figures and Tables

**Table 1:** Synonym list.

Specimen	Test Block/Concrete Cube
High temperature	Elevated temperature
Post-high temperature	After high temperature/post-fire
Compressive strength	Cube compressive strength
Mesoscale model	3D mesoscale model/Numerical model
Residual strength ratio	Strength retention effect
Mortar	Cement paste
Thermo-mechanical response	Mechanical properties under high temperature
Ordinary concrete	Natural aggregate concrete
Peak stress	Ultimate stress
Stress–strain relationship	Constitutive relationship

**Table 2:** Physical properties of CB aggregates.

Physical Properties	Grain Size (mm)	Packing Density (kg/m^3^)	Cylinder Compression Strength (MPa)	Apparent Density (kg/m^3^)	Absorption 1 h (%)
Value	3~15	772	8.0	1750	6.5

**Table 3:** Physical properties of river sand.

Physical Properties	Apparent Density (kg/m^3^)	Packing Density (kg/m^3^)
Value	2540	1450

**Table 4:** Cement major component contents.

Ingredient	SiO_2_	Al_2_O_3_	MgO	Fe_2_O_3_	CaO	Loss on Ignition
Tested cement (%)	19.3	5.3	2.7	2.3	64.2	3.6

**Table 5:** Fly ash major component contents.

Ingredient	SiO_2_	Fe_2_O_3_	CaO	Al_2_O_3_	Loss on Ignition
Tested fly ash (%)	47.2	6.3	1.2	30.2	10.3

**Table 6:** LC25 concrete mix ratio.

*f*_cu,k_ (MPa)	Clinker (kg)	Water Cement Ratio (W/C)	Fine Aggregate (kg)	CB Coarse Aggregate (kg)
LC25	450	0.44	531	693

**Table 7:** LC25 28-day Cubic Compressive Strength.

*f*_cu,k_ (MPa)	28d Compressive Strengths (MPa)	Average Value (MPa)
LC25	26.76418	28.01855
	28.54596	
	28.74551	

**Table 8:** Surface observation results of specimens after high temperature.

Temperature/°C	Cracking	Colour Change	Surface Damage
200	Minor cracks	Grey	-
400	More fine cracks, extending to the middle and increasing in length	White	Chipping, slight detachment of ceramic grains
600	Fine cracks become dense, some areas become flaky	Red	More chipping, aggregate falling off, and broken corners of the specimen

**Table 9:** Results of mass loss rate calculations.

Temperature (°C)	Mass Before High Temperature (After Drying) $m_{1}$ (kg)	Mass After High Temperature $m_{2}$ (kg)	Mass Loss Rate (%)
200	6.425	6.283	2.2%
400	6.224	6.001	3.6%
600	6.021	5.394	10.4%

**Table 10:** Strength of specimens after high temperature.

T (°C)	$f_{cu}$ (Mean ± Standard Deviation) (MPa)	Residual Strength Ratio (Mean ± Standard Deviation) (%)
10	28.02 ± 1.09	-
200	26.44 ± 1.52	94.4% ± 2.1%
400	24.75 ± 0.87	88.3% ± 1.5%
600	15.33 ± 1.68	54.7% ± 2.6%

**Table 11:** Longitudinal and transverse strain contour plots of uniaxial compression at different stress levels in 10 °C specimens.

T/°C	Strain	Initial Loading Stage			$0.3\;f_{cT}$			$0.8\;f_{cT}$			$f_{cT}$		
		με			με			με			με		
10	ε_x_	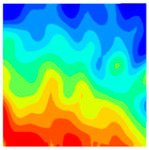 10(a)	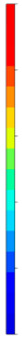	267.6	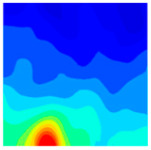 10(b)	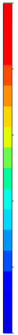	881.0	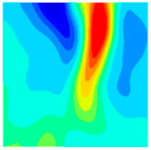 10(c)	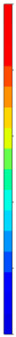	2846.2	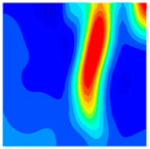 10(d)	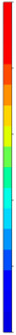	7067.3
				173.4			603.9			2025.8			5478.3
				79.3			326.8			1205.3			3889.4
				−14.9			49.8			384.9			2300.5
				−109.0			−227.3			−435.6			711.6
				−203.1			−504.4			−1256.0			−877.4
	ε_y_	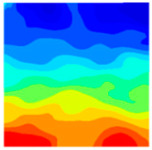 10(a′)	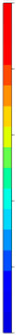	367.6	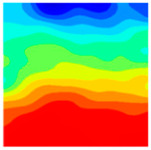 10(b′)	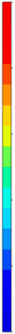	334.9	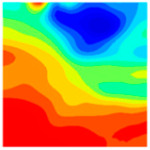 10(c′)	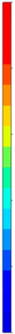	1252.2	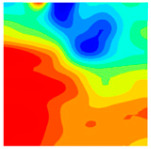 10(d′)	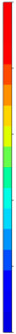	2221.0
				217.7			−4.2			600.6			1392.7
				67.8			−343.2			−50.9			564.5
				−82.0			−682.3			−702.4			−263.8
				−231.9			−1021.4			−1354.0			−1092.1
				−381.8			−1360.4			−2005.5			−1920.4

T—Temperature (°C); ε_x_—Strain in the transverse direction (με); ε_y_—Strain in the longitudinal direction (με); $f_{cT}$—The peak cubic specimen compressive strength at temperature T; 0.3 $f_{cT}\;and$ 0.8 $f_{cT}$ denote 0.3 times and 0.8 times of this strength (MPa).

**Table 12:** Longitudinal and transverse strains of specimens under uniaxial compression at different stress levels after exposure to various high temperatures.

T/°C	Strain	Initial Loading Stage			0.3 *f*_cT_			0.8 *f*_cT_			*f*_cT_		
		με			με			με			με		
200	ε_x_	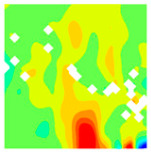	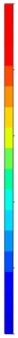	1405.8	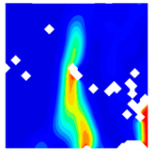	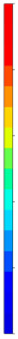	10,844.2	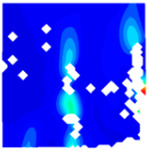	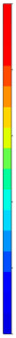	57,715.1	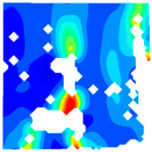	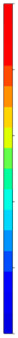	38,232.4
				804.2			8611.9			45,477.1			28,869.4
				202.5			6379.6			33,239.0			19,506.4
				−399.1			4147.2			21,001.0			10,143.4
				−1000.8			1914.9			8763.0			780.3
				−1602.4			−317.5			−3475.1			−8582.7
	ε_y_	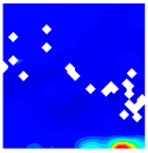	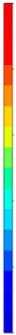	4587.7	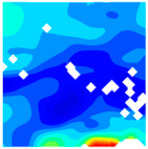	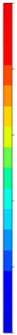	5967.1	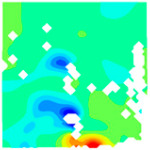	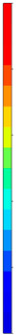	15,646.6	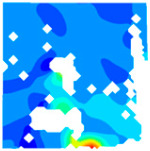	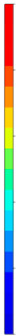	30,658.5
				3484.3			4059.1			9144.0			21,852.7
				2380.8			2151.4			2641.3			13,046.8
				1277.4			243.6			−3861.3			4240.9
				174.0			−1664.3			−10,363.9			−4565.0
				−929.5			−3572.1			−16,866.5			−13,370.8
400	ε_x_	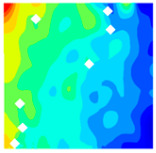	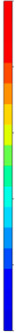	836.3	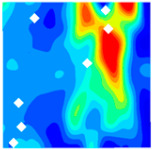	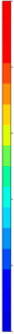	2554.2	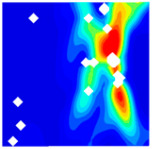	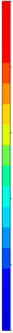	22,563.4	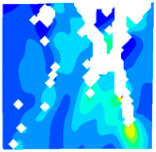	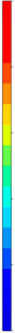	43,331.5
				515.1			1918.6			17,920.4			32,543.4
				193.9			1282.9			13,277.3			21,705.4
				−127.3			647.3			8634.2			10,867.4
				−448.6			11.6			3991.1			29.4
				−769.8			−624.1			−651.9			−10,808.7
	ε_y_	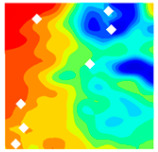	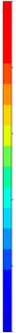	491.5	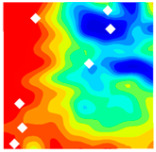	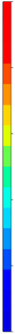	510.7	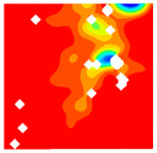	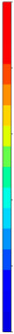	1276.1	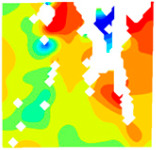	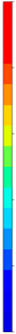	13,213.7
				82.3			−343.2			−2952.7			6312.7
				−327.0			−1197.1			−7181.4			−588.4
				−736.3			−2051.0			−11,410.2			−7489.5
				−1145.5			−2904.9			−15,638.9			−14,390.6
				−1554.8			−3758.8			−19,867.6			−21,291.6
600	ε_x_	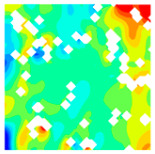	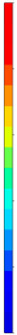	722.3	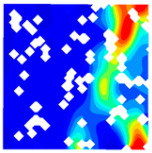	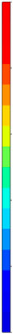	6575.4	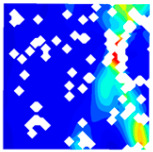	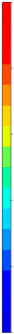	22,828.8	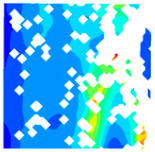	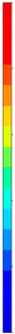	63,674.2
				437.3			5127.2			17,817.7			46,661.5
				152.2			3679.0			12,806.7			29,648.8
				−132.8			2230.9			7795.7			12,636.1
				−417.9			782.7			2784.6			−4376.6
				−702.9			−665.4			−2226.4			−21,389.3
	ε_y_	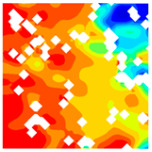	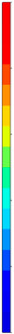	294.0	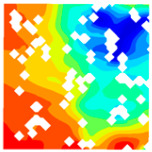	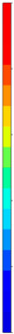	1487.6	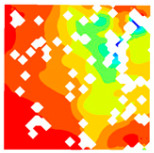	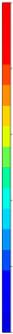	2805.5	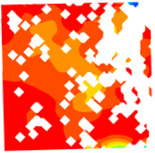	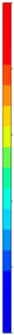	4870.1
				−32.7			95.7			−535.8			−2678.4
				−359.5			−1296.2			−3877.1			−10,226.9
				−686.3			−2688.2			−7218.4			−17,775.3
				−1013.0			−4080.1			−10,559.7			−25,323.8
				−1339.8			−5472.0			−13,901.0			−32,872.3

T—Temperature (°C); ε_x_—Strain in the transverse direction (με); ε_y_—Strain in the longitudinal direction (με); *f*_cT_—The peak cubic specimen compressive strength at temperature T; 0.3 *f*_cT_ and 0.8 *f*_cT_ denote 0.3 times and 0.8 times of this strength (MPa).

**Table 13:** Simulation parameters.

Different Temperatures (°C)	Axial Compressive Strength (MPa)	Peak Strain in Axial Compression	Axial Tensile Strength (MPa)	Peak Strain in Axial Tension	Modulus of Elasticity (MPa)
10	24.63447	0.00204	1.83556	2.04 × 10^−4^	18,700
200	23.39959	0.00399	1.54	0.00532	15,260
400	18.35564	0.03004	1.03	0.00707	7218
600	11.1	0.09998	0.44	0.04138	1234

**Table 14:** Comparison of computational efficiency—3D Mesoscale Model.

Part Type	Mesh Size (mm)	Total Number of Elements	Element Type	Runtime (Hour)
CB Aggregate	3	66,972	C3D10	46
ITZ	4	11,2131	C3D10	
mortar	4	1,251,313	C3D10	

**Table 15:** Comparison of computational efficiency—model after different temperatures.

Temperature (°C)	Mesh Size (mm)	Total Number of Elements	Element Type	Runtime (Hour)
200	5	83,340	C3D8R	6
400	5	83,340	C3D8R	6
600	5	83,340	C3D8R	5.9

## Data Availability

The original contributions presented in this study are included in the article. Further inquiries can be directed to the corresponding author.

## Abbreviations

The following abbreviations are used in this manuscript:

CB	Cold-bonded fly ash aggregate
LWC-CB	A lightweight concrete developed by replacing all natural coarse aggregates with cold-bonded fly ash aggregates
ITZ	Interfacial transition zone
DIC	Digital image correlation
